# Efficient direct ethanol production from cellulose by cellulase- and cellodextrin transporter-co-expressing *Saccharomyces cerevisiae*

**DOI:** 10.1186/2191-0855-3-34

**Published:** 2013-06-24

**Authors:** Ryosuke Yamada, Yuki Nakatani, Chiaki Ogino, Akihiko Kondo

**Affiliations:** 1 Organization of Advanced Science and Technology, Kobe University, 1–1 Rokkodaicho, Nada-ku, Kobe, Hyogo 657-8501, Japan; 2 Department of Chemical Science and Engineering, Graduate School of Engineering, Kobe University, 1-1 Rokkodaicho, Nada-ku, Kobe, Hyogo 657-8501, Japan

**Keywords:** Bioethanol, Cellulase, Yeast, Cellodextrin transporter, Cell surface display, Cellulose

## Abstract

Efficient degradation of cellulosic biomass requires the synergistic action of the cellulolytic enzymes endoglucanase, cellobiohydrolase, and β-glucosidase. Although there are many reports describing consolidation of hydrolysis and fermentation steps using recombinant *Saccharomyces cerevisiae* that express cellulolytic enzymes, the efficiency of cellulose degradation has not been sufficiently improved. Although the yeast *S. cerevisiae* cannot take up cellooligosaccharide, some fungi can take up and assimilate cellooligosaccharide through a cellodextrin transporter. In this study, a *S. cerevisiae* strain co-expressing genes for several cell surface display cellulases and the cellodextrin transporter was constructed for the purpose of improving the efficiency of direct ethanol fermentation from phosphoric acid swollen cellulose (PASC). The cellulase/cellodextrin transporter-coexpressing strain produced 1.7-fold more ethanol (4.3 g/L) from PASC during a 72-h fermentation than did a strain expressing cellulase only (2.5 g/L). Direct ethanol production from PASC by the recombinant *S. cerevisiae* strain was improved by co-expression of cellulase display and cellodextrin transporter genes. These results suggest that cellulase- and cellodextrin transporter-co-expressing *S. cerevisiae* could be a promising technology for efficient direct ethanol production from cellulose.

## Introduction

Dwindling petroleum supplies and growing environmental concerns over petroleum use have led to increasing interest in development of biomass as a feedstock for liquid fuels. Bioethanol produced from biomass resources holds particular promise as an alternative fuel or gasoline extender. Currently, starch-rich biomass serves as the main feedstock used for bioethanol production as it is rapidly hydrolyzed by amylases, resulting in a high yield of glucose. However, lignocellulosic biomasses, such as sugar cane bagasse, corn stover, rice and wheat straw, switchgrass, and poplar, are also regarded as promising starting materials for bioethanol production because they are abundant, inexpensive, renewable, and have favorable environmental properties (Adsul et al. 2011; Sánchez and Cardona 2008). Despite these advantages, lignocellulosic biomass is much more expensive to process than grains because of the need for extensive pretreatment and relatively large amounts of cellulase enzymes for efficient hydrolysis. Therefore, more efficient and cost-effective methods for the degradation and fermentation of cellulosic biomass to ethanol are required.

Efficient degradation of cellulosic biomass requires the synergistic action of the cellulolytic enzymes endoglucanase (EG), cellobiohydrolase (CBH), and β-glucosidase (BGL). Although there are many reports describing relatively low-cost ethanol production from cellulosic material by consolidation of the hydrolysis and fermentation steps using recombinant microorganisms such as the yeast *Saccharomyces cerevisiae* to express cellulolytic enzymes, the cellulose degradation efficiency has not been sufficiently improved (la Grange et al. 2010; Lynd et al. 2005; Yamada et al. 2013). We previously developed a simple method, designated cocktail δ-integration, to optimize the level of cellulase expression for cellulose degradation (Yamada et al. 2010b). In the cocktail δ-integration method, various cellulase expression cassettes are integrated into yeast chromosomes simultaneously in one step, such that strains exhibiting high cellulolytic activity (i.e., strains expressing an optimum ratio of cellulases) can be easily obtained. Use of this method significantly improved the phosphoric acid swollen cellulose (PASC)-degradation activity of cellulase-displaying *S. cerevisiae*, a microorganism that shows tremendous promise for efficient ethanol production from cellulose.

Although yeast such as *S. cerevisiae* cannot take up cellooligosaccharides, some fungi, such as *Neurospora crassa*, can take up and assimilate cellooligosaccharides through a cellodextrin transporter, facilitating rapid growth on cellulose (Tian et al. 2009). Galazka et al. (2010) were the first to report that expression of the *N. crassa* cellodextrin transporter gene enables *S. cerevisiae* to assimilate cellooligosaccharide. The resulting yeast strain could produce ethanol from cellooligosaccharide directly. Furthermore, addition of a cellulase reagent allowed for simultaneous saccharification and fermentation from cellulose, resulting in more efficient ethanol production compared to the wild type strain.

In this study, a *S. cerevisiae* strain that co-expresses genes for cell surface-displayed cellulases and a cellodextrin transporter was constructed in order to improve the efficiency and yield of direct ethanol production from cellulose. Direct ethanol fermentation from PASC was also examined.

## Materials and methods

### Strains and media

Table 1 summarizes the genetic properties of the strains and plasmids used in this study. Briefly, the host for recombinant DNA manipulations was *Escherichia coli* strain NovaBlue (Novagen, Madison, WI, USA). Cellodextrin transporter and intracellular BGL were expressed in the haploid yeast strains *S. cerevisiae* MT8-1 (Tajima et al. 1985) and in previously constructed cellulase surface displaying strain MT8-1/cocδBEC1, which has 8 copies of EGII gene from *Trichoderma reesei*, 2 copies of CBHII gene from *T. reesei*, and 1 copy of BGL1 gene from *Aspergillus aculeatus* (Yamada et al. 2010b). Recombinant yeast strains were screened using synthetic dextrose (SD) medium (6.7 g/L yeast nitrogen base without amino acids [Difco Laboratories, Detroit, MI, USA], and 20 g/L glucose) supplemented with the appropriate amino acids. PASC was prepared from Avicel PH-101 (Fluka Chemie GmbH, Buchs, Switzerland) as amorphous cellulose (Den Haan et al. 2007). Yeast cells were aerobically cultured in yeast/peptone/dextrose (YPD) medium (10 g/L yeast extract, 20 g/L Bacto-peptone [Difco Laboratories], and 20 g/L glucose). Aerobic culture proceeded in 1-L flasks containing 500 mL of medium incubated on a rotary shaker operated at 150 rpm. Ethanol fermentation proceeded in YP medium (10 g/L yeast extract and 20 g/L Bacto-Peptone) supplemented with 20 g/L of either PASC or glucose.

**Table 1:** **Characteristics of the *E. coli *and *S. cerevisiae *strains and plasmids used in this study**

**Strains and plasmids**	**Relevant features**	**Reference**
*E. coli* strain		
Novablue	*endA1 hsdR17(r*_*K12*_^*-*^*m*_*K12*_^*+*^*) supE44 thi-I gyrA96 relA1 lac recA1/F’[proAB*^*+*^*lacI*^*q*^ Z∆M15::Tn10(Tet^r^)]	Novagen
*S. cerevisiae* yeast strains		
MT8-1	MATa ade leu2 his3 ura3 trp1	Tajima et al. (1985)
MT8-1/cocδBEC1	MATa ade leu2 his3 ura3, Cocktail δ-integration of β-glucosidase, endoglucanase, and cellobiohydrolase genes	Yamada et al. (2010b)
MT8-1/δCDTiBGL	MATa ade leu2 his3 trp1, Cocktail δ-integration of intracellular β-glucosidase and cellodextrin transporter genes	This study
MT8-1/cocδBEC1/δCDTiBGL	MATa ade leu2, Cocktail δ-integration of β-glucosidase, endoglucanase, cellobiohydrolase, intracellular β-glucosidase, and cellodextrin transporter genes	This study
Plasmids		
pδU-PGCDT	URA3, Expression of cellodextrin transporter by δ-integration	This study
pδU-PGiBGL	URA3, Expression of intracellular β-glucosidase by δ-integration	This study
pδH-PGiBGL	HIS3, Expression of intracellular β-glucosidase by δ-integration	This study

### Plasmid construction

The polymerase chain reaction primers used in this study were summarized in Table 2. The universal δ-integrative plasmid was constructed as follows. The DNA fragment encoding the promoter sequence of *S. cerevisiae PGK1*, secretion signal sequence of *SAG1*, and 3′ half of the α-agglutinin gene, including the terminator sequence, was prepared by digesting the plasmid PGK406 AG using the restriction enzymes *Xho*I*/Not*I (Yamakawa et al. 2012). The DNA fragment was inserted into the *Sal*I*/Not*I site of the plasmid pδU (Yamada et al. 2010a), and the resulting plasmid was named pδUPGSecAG.

**Table 2:** Polymerase chain reaction primers used in this study

**Primer name**	**Sequence**
CDT(F)	5′-ACAACAAATATAAAAAATGTCGTCTCACGGCTCCCATGACGGGGCCAGCAC-3′
CDT(R)	5′-GTCGGAACCTCCGCCAGCAACGATAGCTTCGGACACATGGCCGTCGGCCT-3′
UiBGL(F)	5′-ACAACAAATATAAAAAGATGAACTGGCGTTCTCTCCTCCTTTCTACCCCTC-3′
UiBGL(R)	5′-GTCGGAACCTCCGCCTTGCACCTTCGGGAGCGCCGCGTGAAGGGGCAGCT-3′
HiBGL(F)	5′-AGCTCCACCGCGGTGCGATTTGGGCGCGAATCCTTTATTTTGGCTTCACCC-3′
HiBGL(R)	5′-TCCACTAGTTCTAGAAGCTTTAACGAACGCAGAATTTTCGAGTTATTAAA-3′
BGL761(F)	5′-CTTCCAGGGCTTTGTGATGTC-3′
BGL858(R)	5′-AGGTGATATCGCCAGGCATT-3′
EGII968(F)	5′-GAACAATCGCCAGGCTATCCT-3′
EGII1043(R)	5′-TTGCTGGCACATGTCTTGTATG-3′
CBHII387(F)	5′-GGTTCCCTCTTTTATGTGGCTAGA-3′
CBHII455(R)	5′-ATGTCGGCCAAGGTTTGCT-3′
CDTI690(F)	5′-GAGCAACTGGTCATGGCGTAT-3′
CDTI761(R)	5′-AAGACGGAGGACATGACGATAAG-3′

The δ-integrative plasmid for expression of cellodextrin transporter gene *CDTI* from *N. crassa* was constructed as follows. The DNA fragment encoding *CDTI* was amplified from *N. crassa* strain NBRC 6068 cDNA using the primers CDT(F) and CDT(R). The fragment was then inserted into the plasmid pδUPGSecAG linearized by *Nhe*I*/Spe*I using In-Fusion cloning kit (Takara Bio, Shiga, Japan). The resulting plasmid was named pδUPGCDTI.

The δ-integrative plasmid for intracellular expression of *BGL1* was constructed as follows. The DNA fragment encoding *A. aculeatus BGL1* gene without its secretion signal sequence was amplified from plasmid pδU-PGAGBGL (Yamada et al. 2010b) using the primers UiBGL(F) and UiBGL(R). The fragment was then inserted into the plasmid pδUPGSecAG *linearized by Nhe*I*/Spe*I using In-Fusion cloning kit. The resulting plasmid was named pδUPGiBGL.

Plasmid pδHPGiBGL, which was used for intracellular expression of *BGL1*, was constructed as follows. The gene cassette for intracellular *BGL1* expression encoding the *S. cerevisiae PGK1* promoter, the *A. aculeatus BGL1* gene, and the *PGK1* terminator was amplified from pδUPGiBGL by PCR using the primers HiBGL(F) and HiBGL(R). The resulting fragment was inserted into the plasmid pδH (Yamada et al. 2010) linearized by *Not*I using In-Fusion cloning kit. The resulting plasmid was named pδHPGiBGL.

### Yeast transformation

Identical amounts (about 20 μg) of each of two comarked δ-integrative plasmids (pδUPGCDT and pδUPGiBGL, which allow expression of *CDT* and intracellular *BGL1*, respectively) were mixed and co-transferred into MT8-1, and the resulting strain was named MT8-1/δCDTiBGL.

A cellulase enzyme expression-optimized strain (MT8-1/cocδBEC1) capable of expressing the enzymes EG, CBH, and BGL was constructed using the cocktail δ-integration method, as described previously (Yamada et al. 2010b). Identical amounts (about 20 μg) of each of two comarked δ-integrative plasmids (pδUPGCDT and pδHPGiBGL, which allow expression of *CDT* and intracellular *BGL1*, respectively) were mixed and co-transferred into MT8-1/cocδBEC1, and resulting strain was named MT8-1/cocδBEC1/δCDTiBGL.

### Ethanol fermentation from cellobiose

Yeast cells were precultured aerobically in YPD medium at 30°C for 24 h, harvested by centrifugation at 1,000 × *g* for 5 min, and then washed twice with distilled water. The wet-cell weight was then determined by harvesting the washed cells by centrifugation at 3,000 × *g* for 5 min and weighing the cell pellet (the estimated dry-cell weight for all strains was approximately 0.15-times the wet-cell weight). The cells were then resuspended to an initial cell concentration of 200 g wet cells/L in 20 mL of YP medium containing 20 g/L of cellobiose. Ethanol fermentation proceeded at 30°C for 96 h with mild agitation in 100-mL closed bottles, each equipped with a siliconized tube and check valve (Sanplatec Corp., Osaka, Japan) as a CO_2_ outlet. Ethanol, glucose, and cellobiose concentrations were measured simultaneously using a high-performance liquid chromatography (HPLC) system (Shimadzu, Kyoto, Japan) equipped with a Shim-pack SPR-Pb column (Shimadzu) maintained at 80°C. The column was eluted with water as the mobile phase at a flow rate of 0.6 mL/min, and the eluate was monitored using a refractive index detector (Shimadzu, RID10A). Culture broth was centrifuged at 14,000 × *g* for 10 min, and a sample of the resulting supernatant was analyzed by HPLC.

### PASC degradation activity

To examine PASC degradation activity, yeast cells were cultivated in YPD medium for 24 h at 30°C, collected by centrifugation at 1,000 × g for 5 min at 4°C, and then washed twice with distilled water. Washed cells were added to a 1-mL solution of 5 g/L PASC in 50 mmol/L sodium citrate buffer (pH 5.0). After the hydrolysis reaction was allowed to proceed for 6 h at 50°C, the supernatant was collected by centrifugation for 5 min at 10,000 × *g* at 4°C to remove cells and debris, and the concentration of the glucose produced was measured using a Glucose CII Test (Wako Pure Chemical Industries, Osaka, Japan). One unit of PASC degradation activity was defined as the amount of enzyme producing 1 μmol glucose/min at 50°C and pH 5.0.

### Quantification of cellulolytic enzyme gene transcription using real-time PCR

The transcription level of each cellulase gene as well as that of the CDT gene was quantified using real-time reverse transcription (RT)-polymerase chain reaction (PCR). Total RNA was isolated from yeast cells cultivated in YPD medium for 24 h at 30°C using a RiboPure Yeast Kit (Ambion, Austin, TX, USA), and cDNA was then synthesized from total RNA using a ReverTra Ace qPCR RT Kit (Toyobo, Osaka, Japan). Real-time RT-PCR using synthesized cDNA as a template was performed using a Stratagene MX3000P qPCR system (Agilent/Stratagene, La Jolla, CA, USA) with four sets of PCR primers (BGL761(F) and BGL858(R), EGII968(F) and EGII1043(R), CBHII387(F) and CBHII455(R), and CDTI690(F) and CDTI761(R)) and Thunderbird SYBR qPCR Mix (Toyobo). The primers BGL761(F) and BGL858(R) were constructed for detecting both intracellular *BGL1* gene transcription and surface displayed *BGL1* gene transcription. Transcription levels of the target genes were normalized to the housekeeping gene *PGK1* using a standard curve method.

### Ethanol fermentation from PASC

Yeast cells were precultured aerobically in YPD medium at 30°C for 72 h, harvested by centrifugation at 1,000 × *g* for 5 min, and then washed twice with distilled water. The wet-cell weight was then determined by harvesting the washed cells by centrifugation at 3,000 × *g* for 5 min and weighing the cell pellet. The cells were then resuspended to an initial cell concentration of 200-g wet cells/L in 20 mL of YP medium containing 20 g/L of PASC. Ethanol fermentation proceeded at 37°C for 96 h with mild agitation in 100-mL closed bottles, each equipped with a siliconized tube and check valve as a CO_2_ outlet. Ethanol concentration was determined using a gas chromatograph (model GC-2010; Shimadzu) equipped with a flame ionization detector and a Durabond Free Fatty Acid Phase (DB-FFAP) column (60 m × 0.25 mm internal diameter; 0.5 μm film thickness; Agilent Technologies, Palo Alto, CA, USA). Helium was used as the carrier gas. The injection volume and split ratio were adjusted to 1 μL and 1:50, respectively. The column temperature was programmed to increase from 40°C to 170°C with a linear gradient of 10°C/min.

## Results

### Co-expression of cellodextrin transporter and β-glucosidase

**Figure 1:**
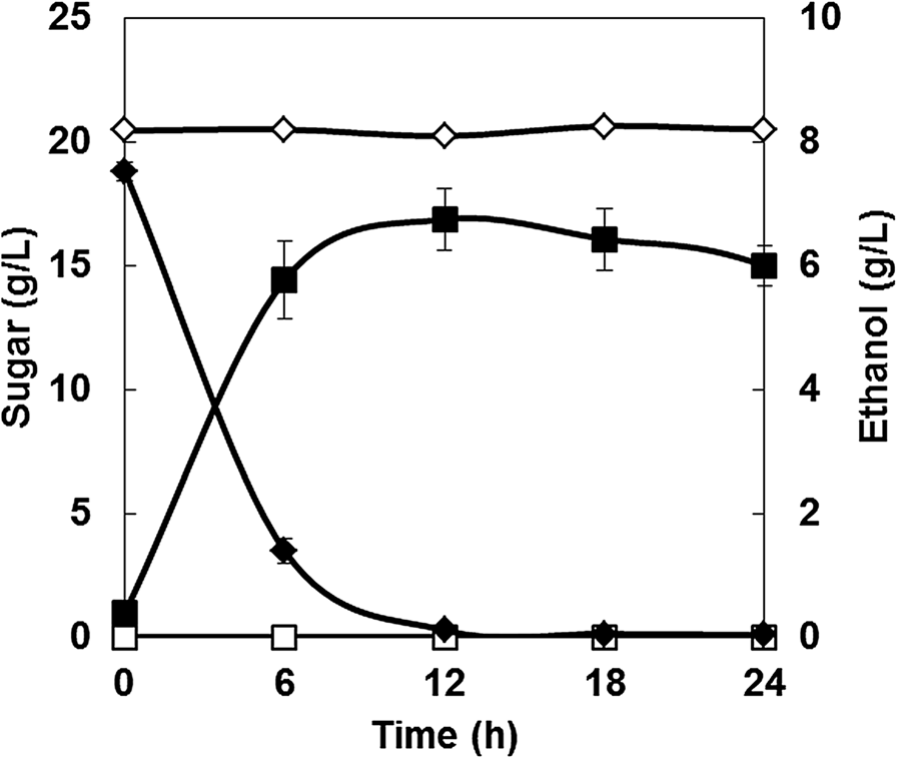
**Time course of ethanol production from cellobiose by engineered strains.** Diamonds = cellobiose; squares = ethanol; open symbols = MT8-1; closed symbols = MT8-1/δCDTiBGL. Data are averages from three independent experiments (error bars represent SE).

Ethanol fermentation from cellobiose was carried out in order to confirm functional intracellular expression of cellodextrin transporter and β-glucosidase. As shown in Figure 1, the cellodextrin transporter/β-glucosidase-co-expressing strain consumed 18.5 g/L of cellobiose and produced 6.4 g/L of ethanol during a 12-h fermentation. The ethanol yield reached 61% of the theoretical yield. In contrast, no detectable ethanol was produced from cellobiose by the wild type strain during a 24-h fermentation. These results confirmed functional intracellular expression of cellodextrin transporter and β-glucosidase gene.

### Cellulase activity of the cellodextrin transporter/cellulase-co-expressing strain

**Figure 2:**
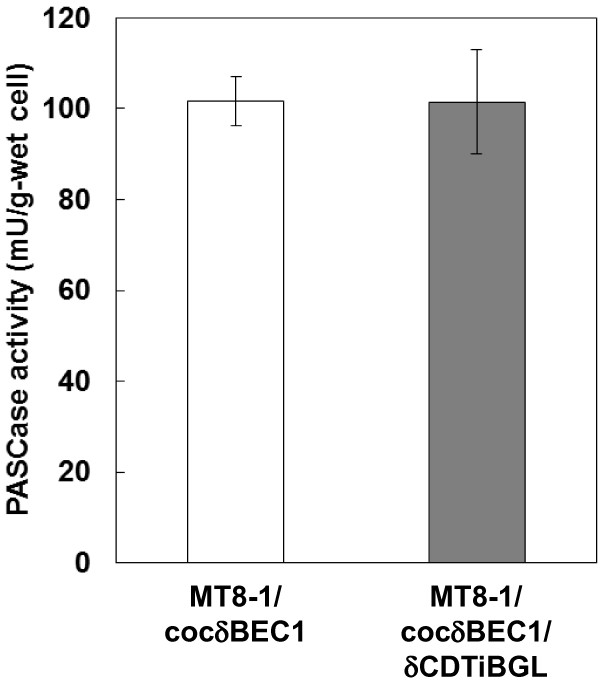
**Degradation of PASC by *CDT*-, intracellular *BGL-*, and cellulase-co-expressing yeast.** White bar = MT8-1/cocδBEC1; gray bar = MT8-1/cocδBEC1/δCDTiBGL. Data are averages from three independent experiments (error bars represent SE).

Degradation of PASC was examined in order to determine the effect of cellodextrin transporter expression on the cellulase activity of the cellodextrin transporter/cellulase-co-expressing stain. As shown in Figure 2, the PASC degradation activity of the cellodextrin transporter/cellulase-co-expressing strain (101.5 mU/g-wet cells) was almost the same as that of the cellulase-expressing strain (101.6 mU/g-wet cells). Thus, the effect of cellodextrin transporter expression on the activity of the cell surface cellulase was negligible.

### Quantification of the transcription level of cellulolytic enzyme genes using real-time RT-PCR

**Figure 3:**
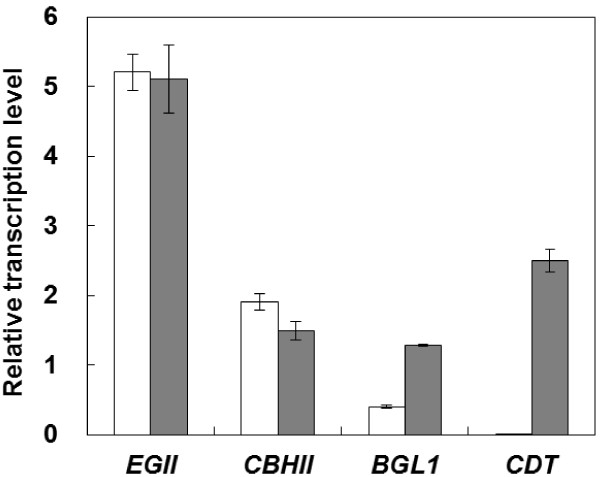
**Relative transcription of cellulase and *CDT *genes.** White bars = MT8-1/cocδBEC1; gray bars = MT8-1/cocδBEC1/δCDTiBGL. Data are averages from three independent experiments (error bars represent SE).

Real-time RT-PCR was carried out in order to confirm the effect of cellodextrin transporter expression on the transcription level of each cellulase gene. As shown in Figure 3, the level of transcription of the *EGII* and *CBHII* genes in the cellulase-expressing strain (5.21 and 1.90, respectively) was almost same as that in the cellodextrin transporter/cellulase-co-expressing strain (5.11 and 1.49, respectively). In contrast, the level of *BGL1* transcription in the cellulase-expressing strain (0.40) was 3.2-fold lower than that in the cellodextrin transporter/cellulase-co-expressing strain (1.29). In addition, the transcription of *CDT* was detected only in the cellodextrin transporter/cellulase-co-expressing strain.

### Ethanol production from PASC by the cellodextrin transporter/cellulase-co-expressing yeast strain

**Figure 4:**
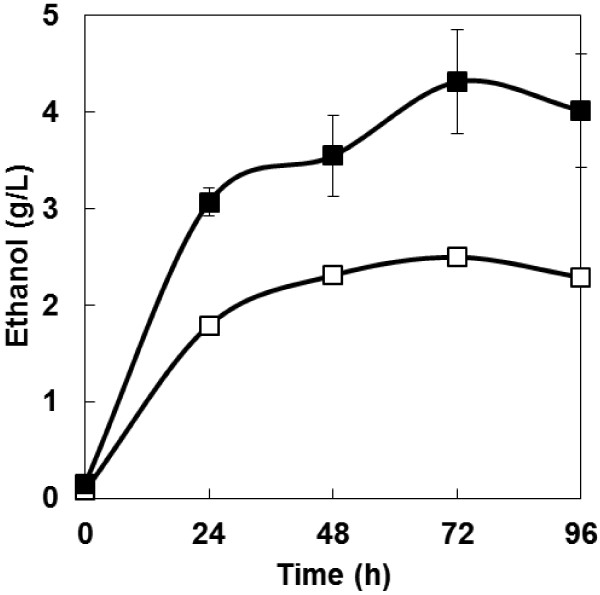
**Time course of ethanol production from PASC by engineered *S. cerevisiae *strains.** Open symbols = MT8-1/cocδBEC1; closed symbols = MT8-1/cocδBEC1/δCDTiBGL. Data are averages from three independent experiments (error bars represent SE).

Direct ethanol production from PASC was carried out in order to evaluate the effect of co-expression of cellulases and the cellodextrin transporter on direct ethanol production from cellulose. As shown in Figure 4, the cellodextrin transporter/cellulase-co-expressing strain produced 4.3 g/L of ethanol from PASC during a 72-h fermentation, a 1.7-fold higher level of production than that of the cellulase-expressing strain (2.5 g/L). Ethanol yield by the cellodextrin transporter/cellulase-co-expressing strain reached 37% of the theoretical yield.

## Discussion

In this study, a *S. cerevisiae* strain co-expressing cellodextrin transporter and cellulase genes was constructed. This strain showed a higher level of ethanol production from PASC than a strain expressing only cellulase genes.

Galazka et al. (2010) were the first to report expression of the *N. crassa* cellodextrin transporter and β-glucosidase genes in *S. cerevisiae*, and subsequent ethanol production from cellobiose by that yeast strain. In this study, *N. crassa* cellodextrin transporter and *A. aculeatus* β-glucosidase, an enzyme with high specific activity (Decker et al. 2000; Takada et al. 1998), were co-expressed in *S. cerevisiae*, and this strain also produced ethanol. Intracellular expressed β-glucosidase activity was not detected in the medium (data not shown). Thus, intracellular expression of β-glucosidase could be effectively achieved. Large amounts of cellulolytic enzymes including cellulase and β-glucosidase are required for efficient degradation of cellulose. Thus, it would be important for efficient direct ethanol production from cellulose to express cellulolytic enzymes, which have high specific activity, such as *A. aculeatus* β-glucosidase used in this study.

As shown in Figure 3, the level of *BGL1* transcription in the cellulase/cellodextrin transporter-co-expressing strain was higher than in the strain expressing only cellulase due to the fact that the genes encoding both the intracellular and extracellular forms of BGL are transcribed in the cellulase/cellodextrin transporter-co-expressing strain. Both intracellular and extracellular forms of *BGL* gene transcription were detected by the same set of primers BGL761(F) and BGL858(R). Although the cellulase/cellodextrin transporter-co-expressing strain expressed cellodextrin transporter and intracellular BGL, the level of *EGII* and *CBHII* transcription did not decrease in comparison to the strain expressing only cellulase. This result indicates the co-expressing strain has sufficient capacity to express more cellulolytic enzyme genes. Thus, increasing the number of integrated genes and/or diploidization could be promising strategies for improving cellulolytic activity in yeast (Yamada et al. 2009).

Although the PASC degradation activity and level of cellulase gene transcription of the cellulase/cellodextrin transporter-co-expressing strain was very similar to that of the strain expressing cellulase only (Figures 2 and 3), it produced more ethanol from PASC than did the strain expressing only cellulase (Figure 4). One explanation for this result could be that product inhibition was avoided. Gruno et al. (2004) and Xiao et al. (2004) reported that the activity of endoglucanase and β-glucosidase enzymes is inhibited by the respective products of these enzymes (cellobiose and glucose, respectively). Although the concentration of cellobiose and glucose were lower than the detection limit in this study, their concentrations could be decreased by introducing a bypass which is cellodextrin assimilating pass way by cellodextrin transporter and intracellular β-glucosidase. Product inhibition could thus be avoided and ethanol productivity improved. This product inhibition avoiding effect is in accordance with a previous study involving amylolytic yeast (Yamakawa et al. 2010). The authors of that report indicated that production of ethanol from raw starch can be improved by introducing a maltose transporter as a bypass. Galazka et al. (2010) used cellodextrin transporter-expressing *S. cerevisiae* for simultaneous saccharification and fermentation from cellulose with addition of cellulase. In contrast, in this study, the cellodextrin transporter was co-expressed with three different cellulase genes. As a result, direct ethanol production from cellulose was improved without the need for addition of cellulase. Thus, the use of *S. cerevisiae* that co-express cellodextrin transporter and cellulase genes is a promising strategy for direct ethanol fermentation from cellulose.

In conclusion, the ability of recombinant *S. cerevisiae* to ferment PASC was improved by engineering the organism to co-express genes encoding cell surface-displayed cellulases and the cellodextrin transporter gene. The ethanol production rate and amount of ethanol produced in this study were low compared to industrial production levels. However, construction of industrial cellulolytic yeast might be achieved in the future by combining techniques such as yeast diploidization with strategies to improve cellulolytic activity, such as optimizing the expression level of each cellulolytic enzyme gene (Yamada et al. 2010b^ 2011^).

## Competing interests

The authors declare that they have no competing interests.

## Authors’ contributions

RY and YN wrote the paper. YN designed and performed the experiments. CO and AK commented and supervised on the manuscript. All authors read and approved the final manuscript.
